# Supplementary material for: Association between oral microbiome and seven types of cancers in East Asian population: a two-sample Mendelian randomization analysis
Source: Front Mol Biosci. 2023 Nov 21;10:1327893. doi: 10.3389/fmolb.2023.1327893 (PMC10702768; doi:10.3389/fmolb.2023.1327893)
Supplement: Supplementary file 1 [file DataSheet1.ZIP › Supplementary Materials/MR plots for tongue/tongue═╝/Prostate cancer/pheno.538_to_prostate cancer_leave_one_out.pdf]

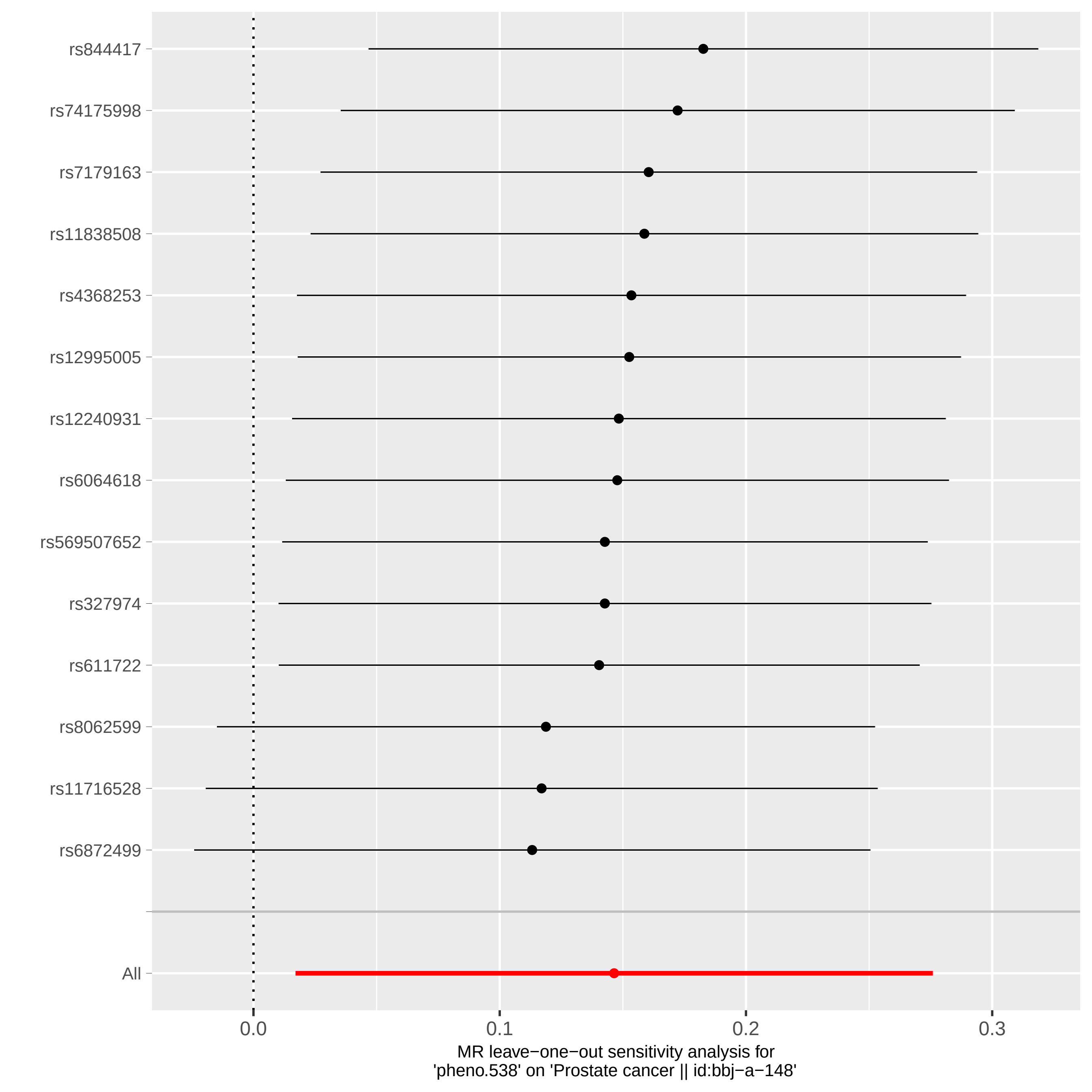
rs844417
rs74175998
rs7179163
rs11838508
rs4368253
rs12995005
rs12240931
rs6064618
rs569507652
rs327974
rs611722
rs8062599
rs11716528
rs6872499
All
0.0
0.1
0.2
0.3
MR leave−one−out sensitivity analysis for
'pheno.538' on 'Prostate cancer || id:bbj−a−148'
